# *ACTN3:* More than Just a Gene for Speed

**DOI:** 10.3389/fphys.2017.01080

**Published:** 2017-12-18

**Authors:** Craig Pickering, John Kiely

**Affiliations:** ^1^School of Sport and Wellbeing, Institute of Coaching and Performance, University of Central Lancashire, Preston, United Kingdom; ^2^Exercise and Nutritional Genomics Research Centre, DNAFit Ltd., London, United Kingdom

**Keywords:** *ACTN3*, genetics, adaptation, recovery, injury, personalized, genetic testing

## Abstract

Over the last couple of decades, research has focused on attempting to understand the genetic influence on sports performance. This has led to the identification of a number of candidate genes which may help differentiate between elite and non-elite athletes. One of the most promising genes in that regard is *ACTN3*, which has commonly been referred to as “a gene for speed”. Recent research has examined the influence of this gene on other performance phenotypes, including exercise adaptation, exercise recovery, and sporting injury risk. In this review, we identified 19 studies exploring these phenotypes. Whilst there was large variation in the results of these studies, as well as extremely heterogeneous cohorts, there is overall a tentative consensus that *ACTN3* genotype can impact the phenotypes of interest. In particular, the R allele of a common polymorphism (R577X) is associated with enhanced improvements in strength, protection from eccentric training-induced muscle damage, and sports injury. This illustrates that *ACTN3* is more than just a gene for speed, with potentially wide-ranging influence on muscle function, knowledge of which may aid in the future personalization of exercise training programmes.

## Introduction

*ACTN3* is a gene that encodes for alpha-actinin-3, a protein expressed only in type-II muscle fibers (North et al., 1999). A common polymorphism in this gene is R577X (rs1815739), where a C-to-T base substitution results in the transformation of an arginine base (R) to a premature stop codon (X). X allele homozygotes are deficient in the alpha-actinin-3 protein, which is associated with a lower fast-twitch fiber percentage (Vincent et al., 2007), but does not result in disease (MacArthur and North, 2004). The XX genotype frequency differs across ethnic groups, with approximately 25% of Asians, 18% of Caucasians, 11% of Ethiopians, 3% of Jamaican and US African Americans, and 1% of Kenyans and Nigerians possessing the XX genotype (Yang et al., 2007; MacArthur et al., 2008; Scott et al., 2010). *ACTN3* genotype is associated with speed and power phenotypes. Yang et al. (2003) reported that elite sprint athletes had significantly higher frequencies of the R allele than controls, a finding that has been replicated multiple times in speed, power and strength athletes (Druzhevskaya et al., 2008; Roth et al., 2008; Eynon et al., 2009; Ahmetov et al., 2011; Cieszczyk et al., 2011; Kikuchi et al., 2016; Papadimitriou et al., 2016; Weyerstraß et al., 2017; Yang et al., 2017), although these findings are not unequivocal (Scott et al., 2010; Gineviciene et al., 2011; Sessa et al., 2011). Whilst Yang et al. (2003) found a trend toward an increased XX genotype frequency in endurance athletes vs. controls, this relationship is less robust, with most studies reporting a lack of association between XX genotype and endurance performance (Lucia et al., 2006; Saunders et al., 2007; Döring et al., 2010; Kikuchi et al., 2016). In addition, whilst Kenyan and Ethiopian endurance runners are highly successful (Wilber and Pitsiladis, 2012), the frequency of the XX genotype within this group is very low at 8% (Ethiopian) and 1% (Kenyan) (Yang et al., 2007). As such, the general consensus is that *ACTN3* X allele likely does not modify elite endurance athlete status (Vancini et al., 2014).

Much of the attention on *ACTN3* has focused on the robust relationship with the R allele and strength/power phenotype, with a number of reviews further exploring this relationship (Eynon et al., 2013; Ma et al., 2013; Ahmetov and Fedotovskaya, 2015). Indeed, a number of papers referenced *ACTN3* as a “gene for speed” (MacArthur and North, 2004; Chan et al., 2008; Berman and North, 2010). However, emerging evidence suggests that this polymorphism may impact a number of other traits, including exercise recovery, injury risk, and training adaptation (Delmonico et al., 2007; Pimenta et al., 2012; Massidda et al., 2017). The purpose of this mini-review is to further explore these potential relationships, as an increased understanding of the role played by *ACTN3* on these traits may lead to improvements in the utilization of genetic information in exercise training.

## *ACTN3* as a modulator of training response

Over the last 20 or so years, the consistent underlying impact of genetics on exercise adaptation has been well explored (Bouchard et al., 2011; Bouchard, 2012). Whilst it is clear that genetics has an undoubted influence on both exercise performance (Guth and Roth, 2013) and adaptation (Mann et al., 2014), fewer studies examine the influence of individual single nucleotide polymorphisms (SNPs) (Delmonico et al., 2007), or a combination of SNPs (Jones et al., 2016), on this process. In this section, we explore the evidence regarding the impact of *ACTN3* on the post-exercise adaptive response.

Following a structured literature search, we found five studies that examined the influence of *ACTN3* on exercise adaptation to a standardized training programme (Table 1). Four of these studied resistance training (Clarkson et al., 2005a; Delmonico et al., 2007; Pereira et al., 2013; Erskine et al., 2014), and one focused on aerobic training (Silva et al., 2015). An additional study (Mägi et al., 2016), monitored changes in VO_2peak_ over a five-year period in elite skiers, with no significant *ACTN3* genotype differences. However, the exercise intervention in this study was not controlled, and so we did not include it within Table 1. There was considerable variation in the findings. For resistance training, two studies reported that the RR genotype was associated with the greatest increase in strength (Pereira et al., 2013) and power (Delmonico et al., 2007) following resistance training. One study reported no effect of *ACTN3* genotype on training adaptations following resistance training (Erskine et al., 2014). Another reported greater improvement in one-repetition maximum (1RM) in X allele carriers compared to RR genotypes (Clarkson et al., 2005a). A further study utilized *ACTN3* within a 15-SNP total genotype score (TGS), finding that individuals with a higher number of power alleles (such as *ACTN3* R) exhibited greater improvements following high-intensity resistance training compared to low-intensity resistance training (Jones et al., 2016). However, because subjects could have the *ACTN3* XX genotype and still be classed as those who would best respond to high-intensity training (due to the possession of a higher number of alleles in other power-associated SNPs), we did not include this study within Table 1.

**Table 1:** Studies examining the interaction between *ACTN3* genotype and exercise adaptation.

**Study**	**Method**	**Sample characteristics**	**Main outcome**
Clarkson et al., 2005a	12 weeks progressive resistance exercise training on non-dominant arm. Progression from 3 sets of 12 repetitions to 3 sets of 6 repetitions, with concurrent increase in load.	602 (355 females) aged 18–40 (*n* = 133 XX genotype).	In females, the X allele was associated with greater absolute and relative improvements in 1RM vs. RR genotypes.
Pereira et al., 2013	12-week high-speed power training programme. Progression from 3 sets of 10 repetitions @ 40% 1RM to 3 sets of 4 repetitions @ 75% 1RM.	139 Older (mean = 65.5 years) Caucasian females (*n* = 54 XX genotype).	RR genotypes exhibited greater performance improvements (maximal strength, CMJ) compared to X allele carriers.
Erskine et al., 2014	9-week unilateral knee extension resistance training programme.	51 previously untrained young males (*n* = 7 XX genotype).	Responses to resistance training were independent of *ACTN3* genotype.
Silva et al., 2015	18-week (3 sessions per week) endurance training programme, comprised primarily of 60-min running, individually controlled by heart rate monitor use.	206 male Police recruits (*n* = 33 XX genotype).	At baseline, XX genotypes had greater VO_2_ measure scores than RR genotypes. Following training, this difference disappeared; i.e., RR had greater improvements than XX.
Delmonico et al., 2007	10-week (3 session per week) unilateral knee extensor strength training comprised of 4–5 sets of 10 repetitions.	155 (*n* = 86 females) older (50–85 years) subjects (*n* = 39 XX genotype).	Change in absolute peak power greater in RR vs. XX (*p* = 0.07) for males. Relative peak power change greater in RR vs. XX (*p* = 0.02).

The variation between studies is likely due to heterogeneity at baseline between genotypes, and differences in exercise prescription. Given the prevalence of the R allele in elite speed-power and strength athletes (Yang et al., 2003; Vincent et al., 2007), it is speculatively considered that R allele carriers would respond best to speed-power and strength training (Kikuchi and Nakazato, 2015). However, as illustrated here, there is perhaps a paucity of data to support this position. Nevertheless, there are some potential molecular mechanisms that could underpin this proposition. Norman et al. (2014) reported that mammalian target of rapamycin (mTOR) and p70S6k phosphorylation was greater in R allele carriers than XX genotypes following sprint exercise. Both mTOR and p70S6k regulate skeletal muscle hypertrophy (Bodine et al., 2001; Song et al., 2005), providing mechanistic support for the belief that hypertrophy, and hence strength and power improvements, should be greater in R allele carriers following resistance training. In addition, Ahmetov et al. (2014) reported that testosterone levels were higher in male and female athletes with at least one R allele compared to XX genotypes. Whilst the direction of this association is not clear, it again supplies a possible mechanism explaining why R allele carriers may experience greater training-induced strength improvements.

A single study examined the impact of this polymorphism on the magnitude of VO_2_ improvements following endurance training (Silva et al., 2015). Here, VO_2_ scores at baseline were greater in XX genotypes, but following training this difference was eliminated, indicating that RR genotypes had a greater percentage improvement following training. The population in this cohort were police recruits. Given that the X allele is potentially associated with elite endurance athlete status (Yang et al., 2003), it is not clear whether these results would be mirrored in elite endurance athletes. Clearly, further work is required to fully understand what relationship, if any, exists between *ACTN3* and improvements in aerobic capacity following training.

## *ACTN3* as a modulator of post-exercise recovery

*ACTN3* R577X has also been associated with exercise-induced muscle damage; here, increased muscle damage will likely reduce speed of recovery, suggesting a potential modifying effect of this polymorphism on between-session recovery. Of the eight studies identified that examined the impact of this polymorphism on post-exercise muscle damage (Table 2), six reported that that the X allele and/or the XX genotype was associated with higher levels of markers associated with muscle damage (Vincent et al., 2010; Djarova et al., 2011; Pimenta et al., 2012; Belli et al., 2017; Del Coso et al., 2017a,b). One study found no effect of the polymorphism (Clarkson et al., 2005b), and one found that RR genotypes experienced a greater exercise-induced reduction in force compared to XX genotypes (Venckunas et al., 2012). An additional study (Del Coso et al., 2017c) examined the impact of *ACTN3* as part of a TGS on creatine kinase (CK) response following a marathon race. Within this TGS, the R allele was considered protective against increased CK concentrations. The results indicated that those athletes with a higher TGS, and therefore greater genetic protection, had a lower CK response to the marathon. Whilst not direct evidence of the R allele's protective effect, as it is possible that the other SNPs used in the TGS conveyed this effect, it nevertheless strengthens the supporting argument.

**Table 2:** Studies examining the interaction between *ACTN3* genotype and exercise recovery.

**Study**	**Method**	**Sample characteristics**	**Main outcome**
Pimenta et al., 2012	Eccentric-contraction based training session.	37 male professional soccer players based in Brazil. (*n* = 9 XX genotype).	Greater creatine kinase (CK) activity in XX genotypes vs. RR.
Clarkson et al., 2005b	50 maximal eccentric contractions of the elbow flexor.	157 male (*n* = 78) and female subjects of various ethnicities (*n* = 115 Caucasians; *n* = 48 XX genotype).	No association of R577X with increases in CK and myoglobin (Mb) following eccentric exercise.
Vincent et al., 2010	4 × 20 maximal single leg eccentric knee extensions.	19 healthy young males (*n* = 10 XX genotype).	XX genotypes had greater peak CK activity post-training compared to RR genotypes, and reported greater increases in muscle pain.
Venckunas et al., 2012	Two bouts of 50 drop jumps.	18 young males (*n* = 9 XX genotype).	RR showed greatest decrease in voluntary force, and slower recovery, compared to XX genotypes.
Djarova et al., 2011	Resting blood sample.	31 South African Zulu males (*n* = 14 Cricketers and *n* = 17 controls). No XX genotypes.	R allele associated with lower CK levels (RR vs. RX).
Del Coso et al., 2017b	Marathon race, pre- and post-race Counter Movement Jump (CMJ).	71 experienced runners (*n* = 8 XX genotype).	X allele carriers had higher CK and Mb levels post-race compared to RR homozygotes. X allele carriers also had a greater reduction in leg muscle power compared to RR genotypes.
Del Coso et al., 2017a	Triathlon competition (1.9 km swim, 75 km cycle, 21.1 km run), pre- and post-race CMJ.	23 healthy, experienced triathletes (*n* = 19 males, *n* = 5 XX genotype).	X allele carriers had a more pronounced jump height reduction compared to RR genotypes. In X allele carriers, there was a tendency toward higher post-race Mb concentrations (*P* = 0.10) and CK concentrations (*P* = 0.06) compared to RR homozygotes.
Belli et al., 2017	37.1 km adventure race (22.1 km mountain biking, 10.9 km trekking, 4.1 km water trekking, 30 m rope course).	20 well trained athletes (*n* = 15 males; *n* = 4 XX genotype).	XX genotypes had higher concentrations of serum Mb, CK, lactate dehydrogenase (LDH) and AST compared to R allele carriers.

The increase in post-exercise muscle damage is likely due to structural changes associated with this polymorphism. Alpha-actinin-3 is expressed only in fast-twitch muscle fibers, and X allele homozygotes are alpha-actinin-3 deficient; instead, they upregulate production of alpha-actinin-2 in these fast-twitch fibers (MacArthur et al., 2007; Seto et al., 2011). Both alpha-actinin-3 (encoded for by *ACTN3*) and alpha-actinin-2 are major structural components of the Z-disks within muscle fibers (Beggs et al., 1992). The Z-disk itself is vulnerable to injury during eccentric contractions (Friden and Lieber, 2001), and knock-out mouse models illustrates these Z-disks are less stable during contraction with increased alpha-actinin-2 concentrations (Seto et al., 2011). A number of the studies in Table 2 exclusively utilized eccentric contractions, whilst others focused on prolonged endurance events that include running, which incorporates eccentric contractions as part of the stretch shortening cycle with each stride (Komi, 2000).

The overall consensus of these studies is that the X allele, and/or the XX genotype, is associated with greater markers of muscle damage following exercise that has an eccentric component; either through direct eccentric muscle action (Vincent et al., 2010), from sport-specific training (Pimenta et al., 2012), or from a competitive event requiring eccentric contractions (Belli et al., 2017; Del Coso et al., 2017a,b). However, there are a number of weaknesses to these studies, potentially limiting the strength of these findings. The overall subject number is modest, with a total of 376 (mean 47) across all eight studies; indeed, the study with the greatest number of subjects, Clarkson et al. (2005b), reported no modifying effect of this polymorphism on post-exercise muscle damage. The total number of XX genotypes was also low, with 85 reported across the studies. This is partly a function of the lower prevalence (~18%) of this genotype, but again the study with the largest number (*n* = 48) of XX genotypes found no effect of this polymorphism (Clarkson et al., 2005b). It is clear that, in order to increase the robustness of this association, further work with greater subject numbers is required.

## *ACTN3* as a modulator of exercise-associated injury risk

We found six studies examining the association between *ACTN3* genotype and sports injury risk (Table 3). Three of these examined ankle sprains (Kim et al., 2014; Shang et al., 2015; Qi et al., 2016), with one each for non-contact injuries (Iwao-Koizumi et al., 2015), professional soccer players (Massidda et al., 2017), and exertional rhabdomyolysis (ER) (Deuster et al., 2013). Whilst ER is strongly related to increased CK following exercise (Clarkson and Ebbeling, 1988; Brancaccio et al., 2010), because it requires medical treatment we classified it as an injury. Of these papers, five reported a protective effect of the R allele and/or the RR genotype against injury (Deuster et al., 2013; Kim et al., 2014; Shang et al., 2015; Qi et al., 2016; Massidda et al., 2017). Specifically, Deuster et al. (2013) found that XX genotypes were almost three times more likely to be ER patients than R allele carriers. Qi et al. (2016) reported a significantly lower frequency of the RR genotype in a group of ankle sprain patients vs. controls. Kim et al. (2014) found that XX genotypes were 4.7 times more likely to suffer an ankle injury than R allele carriers in their cohort of ballerinas. Shang et al. (2015) reported the R allele as significantly under-represented in a cohort of military recruits reporting ankle sprains. Finally, Massidda et al. (2017) demonstrated that XX genotypes were 2.6 times more likely to suffer an injury than RR genotypes, and that these injuries were more likely to be of increased severity. Only one study (Iwao-Koizumi et al., 2015) reported that the R allele was associated with an increased risk (OR = 2.52) of a muscle injury compared to X allele carriers in a female cohort.

**Table 3:** Studies examining the interaction between *ACTN3* genotype and sports injury.

**Study**	**Method**	**Sample characteristics**	**Main outcome**
Iwao-Koizumi et al., 2015	Sports injury data survey.	99 female students (*n* = 34 XX genotype).	R allele associated with an increased odds ratio (OR) of 2.52 of muscle injury compared to X allele.
Deuster et al., 2013	Controls–lower body exercise test. Cases–anonymous blood or tissue sample collected after an exertional rhabdomyolysis (ER) incident.	134 controls and 47 ER patients (*n* = 38 XX genotype)	XX genotypes 2.97 times more likely to be to ER cases compared to R allele carriers.
Qi et al., 2016	Ankle sprain case-control analysis.	100 patients with non-acute ankle sprain vs. 100 healthy controls (*n* = 89 XX genotype).	Significantly lower frequency of RR genotype in ankle sprain group compared to controls (*p* = 0.001).
Kim et al., 2014	Ankle injury case-control analysis.	97 elite ballerinas and 203 normal female adults (*n* = 65 XX genotype).	XX genotypes 4.7 times more likely to suffer an ankle injury than R allele carriers.
Shang et al., 2015	Ankle injury case-control analysis.	142 non-acute ankle sprain patients and 280 physically active controls (*n* = 87 XX genotype). All military recruits.	RR genotype and R allele significantly under-represented in the acute ankle injury group.
Massidda et al., 2017	Case control, genotype-phenotype association study.	257 male professional Italian soccer players and 265 non-athletic controls.	XX players were 2.6 times more likely to suffer a sports injury than RR genotypes. Severe injuries were also more likely in X allele carriers compared to RR genotypes.

Regarding ER, the likely mechanism is similar to that discussed in the post-exercise muscle damage section; increased damage at the Z-disk during exercise. For ankle sprains, the mechanism is potentially related to muscle function. R allele carriers tend to have greater levels of muscle mass (MacArthur and North, 2007), and specifically type-II fibers (Vincent et al., 2007), indicating that both the RX and RR genotypes tend to have increased strength capabilities (Pimenta et al., 2013). For other soft-tissue injury types, again, the decreased potential of damage at the Z-disk likely reduces injury risk. This would be particularly true for eccentric contractions; given the importance of this contraction type in the etiology of hamstring injuries, this could be a further causative mechanism (Askling et al., 2003), alongside that of reduced muscle strength (Yamamoto, 1993).

Alongside the modifying role of *ACTN3* on muscle strength and injury risk, emerging evidence suggests this SNP may also impact flexibility and muscle stiffness. Two studies reported an association between RR genotype and a decreased flexibility score in the sit-and-reach test (Zempo et al., 2016; Kikuchi et al., 2017). Conversely, Kim et al. (2014) reported that XX genotypes had decreased flexibility in the same test. This lack of consensus is largely due to the small total study number, with greater clarity expected as research in the area evolves. It also mirrors the lack of consensus as to whether flexibility increases or decreases risk of injury (Gleim and McHugh, 1997), indicating the complex, multifactorial nature of injuries and their development (Bahr and Holme, 2003).

In summary, it appears that the R allele of *ACTN3* is somewhat protective against injuries. The mechanisms underpinning this are likely varied, and related to a combination of the modifying effects of this SNP on both strength (particularly eccentric strength), exercise-induced muscle damage, and flexibility.

## Discussion

The results of this mini-review indicate that, aside from its established role in sporting performance, the *ACTN3* R577X polymorphism also potentially modifies exercise adaption, exercise recovery, and exercise-associated injury risk. As this polymorphism directly influences both muscle structure and muscle fiber phenotype, this is perhaps unsurprising, and points to the potential use of knowledge of this polymorphism in the development of personalized training programmes. However, it is important to consider the limitations surrounding many of these studies. The subject numbers in the considered studies tended to be low, with large heterogeneity between study cohorts, ranging from untrained subjects to professional sports people, as well as differences in sex. Both of these aspects will impact the study findings; the effect of this polymorphism may be smaller in untrained subjects, for example, whereas in elite, well-trained athletes, who are likely closer to their genetic ceiling, the effect may be greater. The low subject numbers are troubling due to the relatively low XX genotype frequency, which is ~18% in Caucasian cohorts, and even lower in African and African-American cohorts. As such, XX genotypes are considerably under-represented across the considered research.

The above limitations indicate further work is required to fully understand the impact of this polymorphism on these phenotypes. That said, there is some consistency between trials, allowing speculative guidelines to be developed for the use of genetic information in the development of personalized training. XX genotypes potentially have increased muscle damage following exercise that includes an eccentric component (Pimenta et al., 2012; Belli et al., 2017; Del Coso et al., 2017a,b). This information may, consequently, be used to guide between-session recovery, and during the competitive season recovery times post-competition. For example, in an elite soccer club, *ACTN3* genotype could be utilized alongside other well-established markers to determine training intensity in the days following a match, with players genetically predisposed to increased muscle damage either having a longer recovery period, or increased recovery interventions such as cold-water immersion. In addition, recent research has illustrated the positive impact of Nordic Hamstring Exercises on hamstring injury risk (van der Horst et al., 2015), making these exercises increasingly common in professional sports teams. These exercises have a large eccentric component, upon which this polymorphism may have a direct effect. As such, it would be expected that XX genotypes would have increased muscle soreness and damage following these exercises, potentially impacting the timing of their use within a training programme.

Focusing on sporting injuries, the general consensus from the studies found is that the X allele increased the risk of ankle injuries (Kim et al., 2014; Shang et al., 2015; Qi et al., 2016) and general sporting injury (Massidda et al., 2017). Again, this information could guide training interventions. In this case, X allele carriers might undertake increased general strengthening exercises and neuromuscular training targeting injury risk reduction. Furthermore, knowledge of this information could increase athlete motivation to undertake these exercises (Goodlin et al., 2015).

Finally, maximizing the training response is crucial, both to elite athletes looking to improve by fractions of a second, and to beginners looking to decrease their risk of disease. Increasingly, there is evidence that polymorphisms, including *ACTN3* R577X, can impact this adaptive process (Delmonico et al., 2007; Pereira et al., 2013). If further research replicates these early findings, then again, this information could be used in the development of training programmes. Regarding *ACTN3*, at present it appears that R allele carriers potentially exhibit greater increases in strength and power following high-load resistance training (Delmonico et al., 2007). As such, Kikuchi and Nakazato (2015) speculate that R allele carriers should prioritize high-load, low-repetition resistance training if improvements in muscle strength are required, and high intensity interval (HIT) training to specifically elicit improvements in VO_2max_.

## Conclusion

**Figure 1:**
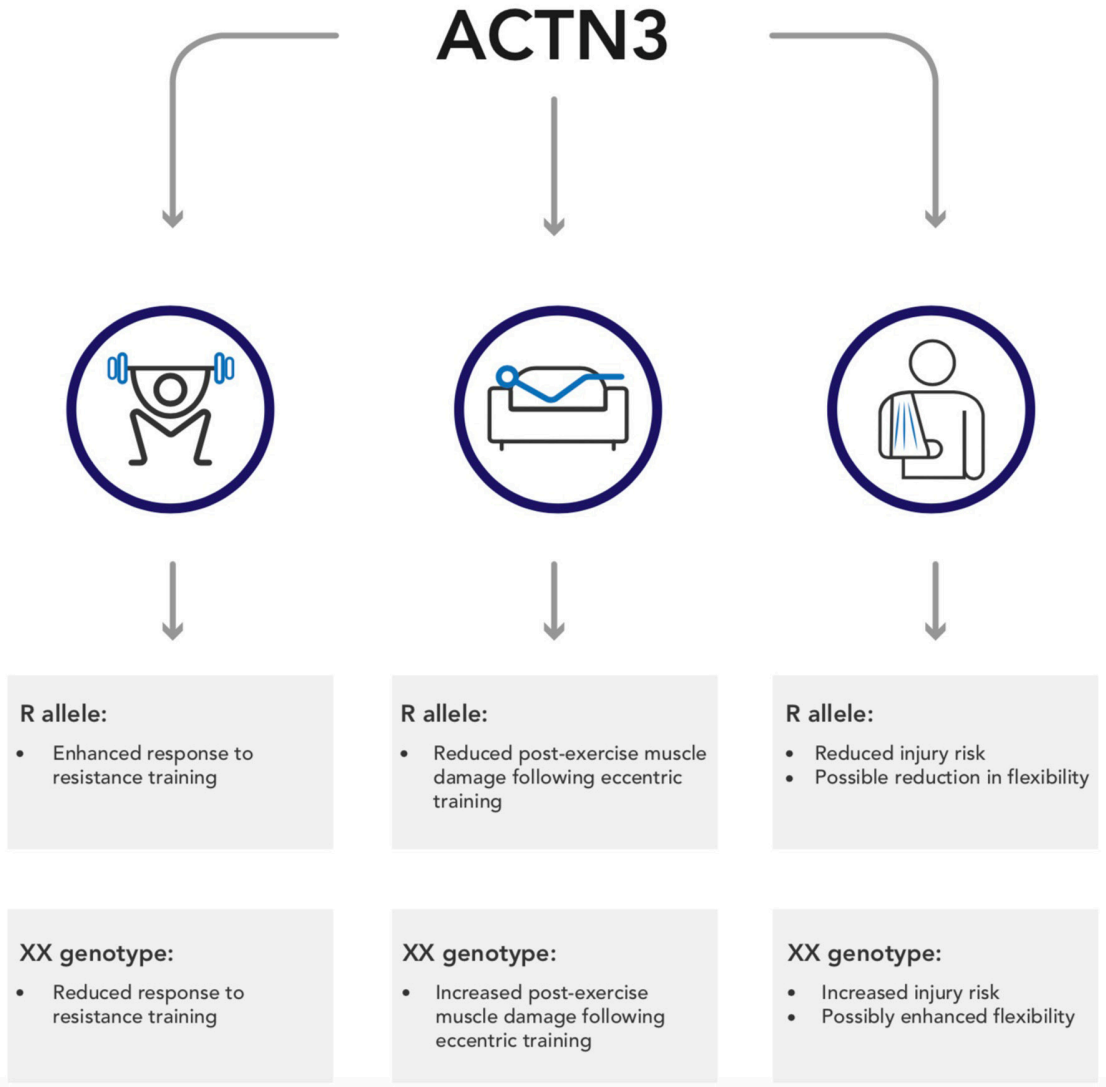
A summary of the potential wider implications of *ACTN3* genotype on outcomes from exercise.

There is a clear, undoubted impact of genetics on both sporting performance and exercise adaptation. In this regard, one of the most well-studied genes is *ACTN3*, which has been reliably shown to impact speed-power and strength phenotypes. However, emerging research indicates that this polymorphism may also impact other exercise associated variables, including training adaptation, post-exercise recovery, and exercise-associated injuries; this research is summarized in Figure 1. This information is important, not just because it illustrates the wide-ranging impact SNPs can have, but also because it represents an opportunity to personalize, and therefore enhance, training guidelines. At present, there are no best-practice guidelines pertaining to the use of genetic information in both elite sport and the general public. However, sports teams have been using genetic information for over 10 years (Dennis, 2005), and continue to do so. Consequently, the development of these guidelines represents an important step from lab to practice. Clearly, further research is required to fully develop these guidelines, and at present such information is speculative. Nevertheless, the use of genetic information represents an opportunity to enhance training prescription and outcomes in exercisers of all abilities.

## Author contributions

CP: Conceived the idea for this manuscript, and wrote the initial draft. JK: Provided feedback on the initial draft, and made valuable changes to the manuscript, as well as providing direction. All the authors made contributions in drafting the manuscript and have approved the final version.

### Conflict of interest statement

CP is an employee of DNAFit Ltd., a genetic testing company. He received no payment for the production of this article, which was completed as part of his Professional Doctorate studies at the University of Central Lancashire. The other author declares that the research was conducted in the absence of any commercial or financial relationships that could be construed as a potential conflict of interest.
